# Impact of Healthcare Associated Infections on Survival and Treatment Outcomes Among End Stage Renal Disease Patients on Renal Replacement Therapy

**DOI:** 10.3389/fphar.2021.707511

**Published:** 2021-08-10

**Authors:** Saad Hanif Abbasi, Raja Ahsan Aftab, Pauline Siew Mei Lai, Soo Kun Lim, Ruwaida Nur Zainol Abidin

**Affiliations:** ^1^ School of Pharmacy, Taylor’s University, Subang Jaya, Malaysia; ^2^ Department of Primary Care Medicine, Faculty of Medicine, University of Malaya, Kuala Lumpur, Malaysia; ^3^ Department of Medicine (Division of Nephrology), Faculty of Medicine, University of Malaya, Kuala Lumpur, Malaysia; ^4^ Department of Pharmacy, Hospital Serdang, Kajang, Malaysia

**Keywords:** healthcare associated infections, end stage renal disease (ESRD), renal replacement therapy (RRT), peritoneal dialysis (PD), hemodialysis (HD), treatment outcome

## Abstract

**Background:** Due to frequent hospitalizations, complex dialysis procedures and immune compromising effects of end stage renal disease (ESRD), patients on dialysis are more prone to healthcare associated infections (HCAIs). **Objective:** To study the impact of HCAIs on survival and treatment outcomes among ESRD patients on renal replacement therapy (RRT). **Methodology:** A multicenter, retrospective study was conducted from June to December 2019 at two public hospitals of Malaysia. ESRD patients with minimum of 6 months on RRT were included, while pregnant patients and patients <18 years were excluded. Multinomial logistic regression was performed to identify risk factors associated with unsuccessful treatment outcomes. Kaplan Meier analysis was performed to study the survival. **Results:** A total of 670 records were examined, of which 400 patients were included as per the inclusion criteria. The mean survival time of patients without HCAIs [22.7 (95%CI:22.1–23.2)] was higher than the patients with HCAIs [19.9 (95%CI:18.8–20.9)]. Poor survival was seen in patients with >2 comorbidities, >60 years of age, low hemoglobin concentration and high C-reactive protein levels. The most frequent treatment outcome was cured [113 (64.9%)], followed by death [37 (21.3%)] and treatment failure [17 (9.8%)]. Advancing age, and low hemoglobin concentration were independent risk factors associated with death, while recurrent HCAIs, use of central venous catheters, and low serum sodium levels were risk factors for treatment failure. **Conclusion:** The high burden of HCAIs is a profound challenge faced by patients on RRT, which not only effects the treatment outcomes but also contributes substantially to the poor survival among these patients.

## Introduction

According to an estimate, the global prevalence of end stage renal disease (ESRD) patients undergoing maintenance hemodialysis is increased by 1.7 times from 1990 to 2010 (Thomas et al., 2015). Due to frequent hospitalizations, complex dialysis procedures and immune compromising effects of ESRD, patients on dialysis are more prone to healthcare associated infections (HCAIs) (Donlon et al., 2011; Albuquerque et al., 2014). Moreover, HCAIs are among the major causes of increased morbidity and mortality among these patients (Gupta et al., 2013). According to the United States renal data system, sepsis mortality was 100–300 times higher for chronic dialysis patients compared to general public (Sarnak and Jaber, 2000). Furthermore, almost 20% of patients on renal replacement therapy (RRT) had at least one episode of septicemia (Sarnak and Jaber, 2000). The recent report of Malaysian dialysis and transplant registry report (Ghazali et al., 2016) suggested that sepsis accounts for 28% of all causes of deaths among patients on dialysis (Ghazali et al., 2016).

Antibiotic treatment failure, which is one of the representatives of antibiotic resistance is increased alarmingly over the past few decades and World Health Organization has declared this a global public health crisis (World Health Organization, 2012). It is still unclear whether the differences in mortality and treatment outcomes among dialysis patients result from differences in patient- and facility related factors, inappropriate, suboptimal, or delayed antibiotic therapy, various complications and comorbid conditions, or other factors. There is a need to better characterize and understand the epidemiology and impact of HCAIs on antibiotic treatment outcomes and patients’ survival, which is not simple owing to different infection types, pathogens’ characteristics and large number of antibiotic treatment options available. Only limited information is available on the factors that contribute to poor clinical outcomes and survival among the high risk ESRD population. The present study was performed to determine the impact of HCAIs on treatment outcomes and survival among ESRD patients on RRT.

## Methodology

### Study Setting and Study Design

A multicenter, cross sectional, retrospective study was conducted at two study sites in Malaysia, including the University Malaya Medical Centre, and Serdang Hospital. UMMC is a tertiary hospital, located in Kuala Lumpur, Malaysia. The UMMC services over 900 inpatients and approximately 1,500 outpatients daily. On average, 80–90 patients undergo hemodialysis (HD) in this hospital. Hospital Serdang is a tertiary hospital situated in the Sepang District in Selangor. It has 100 HD patients and more than 300 patients on peritoneal dialysis (PD). Both hospitals are government funded.

### Patient Population

ESRD patients who were on either HD or PD for a minimum period of 6 months were included, while patients who were pregnant, <18 years and those who have undergone kidney transplantation were excluded. Data of patients who were diagnosed with ESRD (defined as those classified with the international classification of diseases (ICD)-10-CM code N18.6 for ESRD (Wang and Hoy, 2013)) from 2015 to 2019 were extracted from the hospital’s medical records computerized system. All patients were then screened to see if they fit the study’s inclusion/exclusion criteria. The medical records and laboratory data were reviewed using the hospital’s total hospital information system (THIS) to confirm the diagnosis of any HCAI. All data were collected from the time of initiation of dialysis for each patient till 2019. A data collection form was used to retrieve patient demographics and clinical information.

The sample size required was 400 case records (with 80% power and 95% confidence interval (Raosoft. Sample size calc, 2004). To reduce the risk of selection bias, a simple random sampling technique—a research randomizer (Research Randomizer, 1997) was used. Research randomizer utilizes the total number of patients and the sample size to generate random numbers. Each number was noted, and the patient associated with that number on the list was selected.

### Criteria for the Selection of Healthcare Associated Infections

For the purpose of this study, the modified definition from Friedman et al. (2002) was adopted as the initial definition of HCAIs (Friedman et al., 2002), where HCAI is defined as “an infection present at the time of hospital admission or within 48 h of admission, resulting from medical care or treatment in a hospital, primary healthcare setting, nursing home, or patient’s home”. An infection was said to be healthcare associated, if the patient had fulfilled any of the following criteria (Friedman et al., 2002), including 1)received any intravenous therapy, wound care or specialized nursing care at home or through a healthcare agency or had self-administered intravenous therapy in 30 days before the infection, 2)had a hospital visit or received intravenous chemotherapy in the previous 30 days or 3)was hospitalized in an acute care hospital for 2 or more days in the previous 90 days.

Following definitions of treatment outcomes were used in the study (Zelenitsky et al., 2003; Wen et al., 2018; Çilli et al., 2018):

Clinical cure (successful treatment outcome) was defined as resolution of clinical signs and symptoms within 30 days of infection or have negative blood culture after completion of therapy. Death (unsuccessful treatment outcome) was defined as mortality due to any cause during treatment within 2 days (early mortality) or within 2–30 days (late mortality) of a positive blood culture. Treatment failure (unsuccessful treatment outcome) indicated persistent infection or unimproved patient’s clinical status and the need to switch to another antibiotic regimen after 3 days of initial treatment. Finally, defaulted or transferred out patients were those whose treatments were interrupted for any reason or those who were transferred to another healthcare facility before completion of the therapy.

### Study Endpoints

The primary endpoint of the study was the 24 months survival (Etikan et al., 2017) of patients from November 2017 till the completion of medical records viewing, whereas the secondary endpoint was mortality due to any cause during this period. All transferred patients and those who could not be followed up through record viewing were tracked using their contact numbers to get their final status. Any patient with an unidentified final status was not included in the survival analysis. Patients were divided during survival analysis as complete follow-up (alive), primary end point reached (alive), death (dead), transferred out (alive/dead).

### Ethical Considerations

Ethical approval was obtained from the University Malaya Medical Research Ethics Committee (MREC ID NO: 2019320–7245) and the National Malaysian Research Registry (NMRR-19–777–47089) before the initiation of study.

### Statistical Analysis

Data were coded into nominal, ordinal and continuous variables using the Statistical Package for Social Sciences version 20.0 (SPSS Inc., Chicago, IL, United States). Kolmogorov–Smirnov test was used to assess the normality of data. Both descriptive and inferential statistics were applied to achieve the study objectives. A descriptive analysis of patients’ data was conducted to examine the variables of interest. All categorical variables were presented as absolute frequencies and percentages, while median and interquartile range were used to present continuous variables as normality could not be assumed. Both Chi-square and Fischer’s exact test were used to assess all associations involving categorical variables. These significantly associated variables were included in regression analysis. As the dependent variable was of categorical type with more than two levels, multinomial logistic regression was used to analyze the relationship between independent variables and unsuccessful treatment outcomes. Both univariate and multivariate multinomial logistic regression were used to identify independent risk factors associated with unsuccessful treatment outcomes. Presence and absence of HCAIs were analyzed against the patient’s status using Kaplan Meier analysis to study the survival among two patient groups (with and without HCAIs). A *p*-value of <0.05 was considered to be statistically significant.

## Results

Out of 670 patient records identified during database search, 400 patients were included as that fitted the inclusion criteria. Fifty seven out of 400 patients were defaulted or transferred out and their final status was unknown, hence they were excluded from survival analysis. Of the remaining 343 participants, there were 142(41.3%) and 201(58.7%) patients with and without HCAIs, respectively. There were significantly a greater number of deaths [53(37.3%)] in the group of patients with HCAIs, compared to 22(10.9%) deaths in the group without HCAIs (*p*-value <0.001). Baseline characteristics of participants in both the groups are shown in Table 1.

**TABLE 1:** Matching criteria for survival analysis: Baseline characteristics of interest in patients with and without healthcare associated infections

	Total no. of ESRD patients on RRT (N = 400)	Patients with HCAIs (*n*= 142)	Patients without HCAIs (*n* = 201)	*p*-value^a^
**Median age in years (IQR)**	62 (18)	61.5 (17)	61 (20)	**0.001**
**Gender**				**0.028**
Male	219 (54.8 %)	66 (46.5%)	118 (58.7%)	
Female	181 (45.3%)	76 (53.5%)	83 (41.3%)	
**Age group**				**0.257**
18–40 years	48 (12%)	11 (15.9%)	32 (7.7%)	
41–60 years	120 (30%)	47 (30.8%)	62 (33.1%)	
>60 years	232 (58%)	84 (53.2%)	107 (59.2%)	
**Body mass index (kg/m** ^**2**^ )* (n = 324)				0.134
Under weight (Below 18.5)	28 (8.6%)	11 (9%)	13 (9%)	
Healthy or normal (18.5—24.9)	130 (40%)	48 (39.3%)	62 (38.3%)	
Overweight (25—29.9)	107 (33%)	34 (27.9%)	63 (38.9%)	
Obese (More than 30)	59 (18.2%)	29 (23.8%)	24 (14.8%)	
**Race**				0.434
Malay	207 (51.8%)	83 (58.9%)	101 (50.2%)	
Chinese	135 (33.8%)	40 (28.4%)	72 (35.8%)	
Indian	55 (13.8%)	17 (12.1%)	27 (13.4%)	
Others^b^	3 (0.75%)	1 (0.7%)	1 (0.5%)	
**Comorbidities**				
Diabetes	260 (65%)	105 (73.9%)	118 (68.7%)	**0.004**
Hypertension	360 (90%)	131 (92.3%)	177 (88.1)	0.227
Hyperlipidemia	76 (19%)	31 (21.8%)	33 (16.4%)	0.209
Ischemic heart disease	94 (23.5%)	30 (21.1%)	53 (26.4%)	0.306
Congestive heart failure	26 (6.5%)	7 (4.9%)	5 (2.5%)	0.246
Cerebrovascular accident	16 (4%)	12 (8.5%)	10 (5%)	0.263
Hyperparathyroidism	23 (5.8%)	4 (2.8%)	10 (5%)	0.412
Hypoparathyroidism	17 (4.3%)	4 (2.8%)	2 (1%)	0.236
Hepatitis B	22 (5.5%)	4 (2.8%)	1 (0.5%)	0.097
**No. of comorbidities**				**0.036**
2 or less	181 (45.3%)	53 (37.3%)	99 (49.3%)	
More than 2	219 (54.8%)	89 (62.7)	102 (50.7%)	
**No. of medications patient was taking**				0.914
Less than 5	4 (1%)	2 (1.4%)	2 (1%)	
5 to 10	267 (68%)	95 (66.9%)	135 (68.5%)	
More than 10	124 (31%)	45 (31.7%)	60 (30.5%)	
**No. of years on dialysis**				
2 or less	163 (40.8%)	58 (40.8%)	71 (35.3%)	
More than 2	237 (59.2%)	84 (59.2%)	130 (64.7%)	
**Type of dialysis**				0.228
Hemodialysis	222 (55.5%)	81 (57%)	101 (50.2%)	
Peritoneal dialysis	178 (44.5%)	61 (43%)	100 (49.8%)	
**Type of access**				
Arterioventricular fistula (AVF)	104 (26%)	24 (17%)	70 (34.8%)	
Central venous catheter (CVC)	75 (18.8%)	32 (27.7)	21 (10.4)	
Tenchkoff catheter	170 (42.5%)	57 (40.4%)	95 (47.3%)	
Multiple accesses^c^	51 (12.8%)	28 (19.9%)	15 (7.5%)	
**Vital status**				**<0.001**
Alive	268 (67%)	89 (62.7%)	179 (89.1%)	
Dead	75 (18.8%)	53 (37.3)	22 (10.9%)	
Status unknown	57 (14.3%)			
**History of HCAI**	174 (43.5%)			

Bold values mean significant *p*-values.

* Incomplete patient records.

a Chi-square test.

b Thai and Indonesian.

c More than one type of dialysis access used during renal replacement therapy, IQR, interquartile range.

**FIGURE 1:**
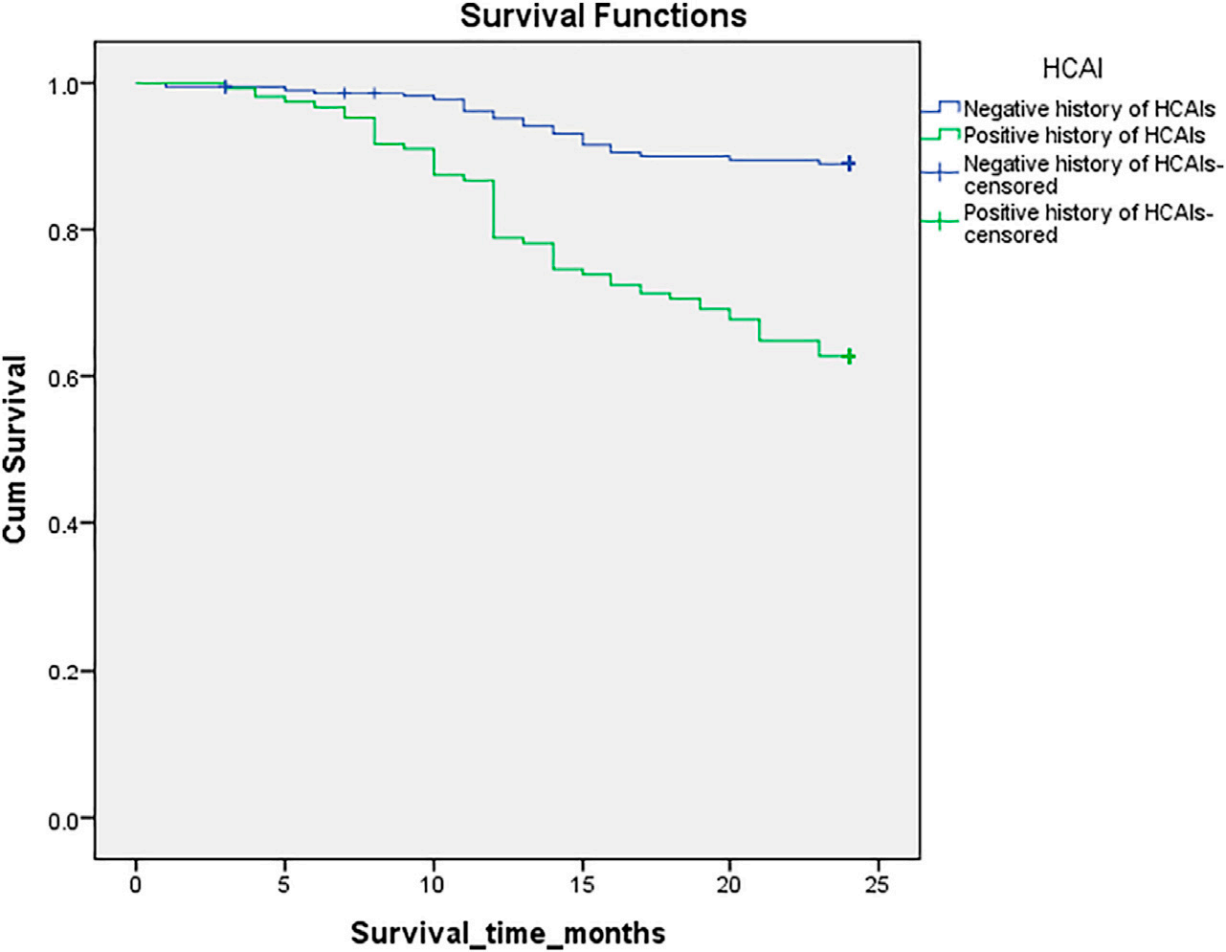
Kaplan-Meier survival analysis in patients with and without healthcare associated infections. Cum survival; Cummulative survival.

The total number of deaths during the 24 months follow-up period was n = 53 (37.3%) for patients’ group with HCAIs and *n* = 22(10.9%) for the group without HCAIs. The mean survival time of patients in the group without HCAIs [22.7(95%CI:22.1–23.2)] was higher than the group with HCAIs [19.9(95%CI:18.8–20.9)] and was found to be statistically significant (*p*-value <0.001; Figure 1). During the study, a significantly better survival (*p*-value = 0.001) was observed among patients aged 18–40 years [22.8(95%CI:21.7–23.9)] and 41–60 years [22.5(95%CI:21.7–23.3)] as compared to patients aged >60 years [20.7 (95%CI:19.8–21.5)]. Survival trends of study population are shown in Table 2.

**TABLE 2:** Survival trends of study participants through Kaplan-Meier survival analysis.

Variables	Total no. of participants, (N = 342)	Time, months	Death, n	Mean survival time, months (SE)	95% CI	*p*-value
**HCAIs**						**<0.001**
Postive history	**142**	24 months	**53**	19.9 (0.52)	18.8–20.9	
Negative history	201	24 months	22	22.7 (0.27)	22.1–23.2	
**Gender**						**0.303**
Male	184	24 months	44	21.1 (0.41)	20.3–21.9	
Female	159	24 months	31	21.9 (0.37)	21-22.1	
**Age group**						**0.001**
18–40 years	43	24 months	4	22.8 (0.56)	21.7–23.9	
41–60 years	109	24 months	15	22.5 (0.39)	21.7–23.3	
>60 years	191	24 months	56	20.7 (0.425)	19.8–21.5	
Body mass index (kg/m2)* (n = 284)						0.282
Under weight (Below 18.5)	24	24 months	8	19.7 (1.2)	17.2–22.3	
Healthy or normal (18.5—24.9)	110	24 months	27	21.6 (0.4)	20.7–22.6	
Overweight (25—29.9)	97	24 months	17	22 (0.4)	21.1–22.9	
Obese (More than 30)	53	24 months	14	20.9 (0.7)	19.3–22.4	
**Race**						0.357
Malay	184	24 months	41	21.6 (0.36)	20.9–22.3	
Chinese	112	24 months	26	21.2 (0.53)	20.1–22.2	
Indian	44	24 months	7	22.2 (0.66)	20.9–23.5	
Others	2	24 months	1	14 (7)	0.1–27.8	
**Type of dialysis**						0.319
Hemodialysis	182	24 months	35	21.5 (0.39)	20.7–22.3	
Peritoneal dialysis	161	24 months	40	21.5 (0.39)	20.7–22.3	
**No. of years on dialysis**						0.657
2 or less years	129	24 months	30	21.3 (0.49)	20.3–22.2	
>2 years	214	24 months	45	21.7 (0.33)	21-22.3	
**Type of vascular access**						
Arterioventricular fistula (AVF)	94	24 months	12	22 (0.54)	21-23.1	
Central venous catheter (CVC)	53	24 months	15	20.6 (0.79)	19-22.1	
Tenchkoff catheter	152	24 months	39	21.4 (0.41)	20.6–22.2	
Multiple accesses	43	24 months	9	21.9 (0.68)	20.5–23.2	
**No. of comorbidities**						**0.005**
1 or 2	152	24 months	23	22.5 (0.31)	21.9–23.1	
More than 2	191	24 months	52	20.7 (0.42)	19.9–21.6	
**Diabetes**						0.271
Yes	223	24 months	53	21.4 (0.34)	20.8–22.1	
No	120	24 months	22	21.6 (0.48)	20.7–22.6	
**Hypertension**						**0.296**
Yes	308	24 months	70	21.4 (0.29)	20.89–22	
No	35	24 months	5	22.2 (0.80	20.6–23.8	
**Hyperlipidemia**						**0.193**
Yes	64	24 months	18	21 (0.64)	19.7–22.2	
No	279	24 months	57	21.6 (0.31)	21.22.2	
**Ischemic heart disease**						**0.489**
Yes	83	24 months	20	20.8 (0.67)	19.5–22.1	
No	260	24 months	55	21.7 (0.3)	21.1–22.3	
**Cerebrovascular accident**						0.607
Yes	22	24 months	4	22.5 (0.77)	20.9–24	
No	321	24 months	71	21.4 (0.29)	20.9–22	
No. of medicicines patient was taking* (n = 339)						**0.035**
Less than 5	4	24 months	3	18 (2.3)	13.3–22.6	
5 to 10	230	24 months	51	21.4 (0.34)	20.8–22.1	
More than 10	105	24 months	21	21.7 (0.49)	20.7–22.6	
**TWBCs**						**<0.001**
High	110	24 months	41	19.4 (0.61)	18.2–20.6	
Normal	220	24 months	27	22.7 (0.25)	22.2–23.2	
Low	13	24 months	7	18.6 (1.7)	15.2–22	
**Hemoglobin**						**0.004**
Normal	70	24 months	6	22.9 (0.45)	22-23.8	
Low	273	24 months	69	21.1 (0.32)	20.5–21.8	
RBCs* (n = 330)						**0.054**
Normal	60	24 months	7	22.6 (0.54)	21.5–23.7	
Low	270	24 months	63	21.4 (0.31)	20.8–22	
Urea* (n = 341)						0.060
High	299	24 months	71	21.4 (0.3)	20.8–21.9	
Normal	42	24 months	4	22.6 (0.6)	21.3–23.9	
**Sodium**						**0.001**
Normal	228	24 months	38	22.2 (0.3)	21.6–22.8	
Low	115	24 months	37	20.2 (0.55)	19.1–21.3	
Total protein* (n = 312)						**0.001**
Normal	178	24 months	1	23.5 (0.45)	22.6–24.4	
Low	134	24 months	74	21.2 (0.31)	20.6–21.8	
Random blood sugar* (n = 284)						**<0.001**
High	106	24 months	33	20.6 (0.56)	19.5–21.7	
Normal	178	24 months	24	22.4 (0.31)	21.8–23.1	
HbA1c range* (n = 310)						0.196
High	**169**	24 months	42	21.3 (0.4)	20.6–22.1	
Normal	**141**	24 months	26	21.7 (0.4)	20.8–22.5	
**C-reactive protein**						**<0.001**
High	**212**	24 months	66	20.5 (0.4)	19.7–21.3	
Normal	**127**	24 months	9	23.2 (0.28)	22.6–23.8	

* Incomplete patient records.

HCAI, healthcare associated infection; TWBCs, total white blood cells; RBCs, red blood cells. Bold values mean significant *p*-values.

Cox regression was applied for predictors associated with survival among study population. Group of patients with HCAIs (HR 1.4) had poor survival and 1.4-folds increased chances of death than group of patients without HCAIs, but the result was statistically nonsignificant (*p* = 0.288). Moreover, poor survival was seen among patients with >2 comorbidities, >60 years of age, Low Hb concentration and high CRP levels during 24-months survival analysis. However, during multivariate regression analysis, factors such as more than 2 comorbidities, above 60 years of age, low hemoglobin levels and high C-reactive protein concentration were not found to be significantly associated with survival among ESRD patients with or without HCAIs **(** Table 3 **)**.

**TABLE 3:** Univariate and multivariate Cox regression analysis of study participants’ predictors for survival.

Variable	Univariate cox regression analysis			Multivariate cox regression analysis		
	Hazard ratio (HR)	Standard error (SE)	*p*-value	Hazard ratio (HR)	Standard error (SE)	*p*-value
**History of HCAI**	1.4	0.3	0.288	-	-	-
**>2 comorbidities**	1.8	0.3	**0.053**	1.0	0.3	0.932
**>60 years of age**	1.9	0.3	**0.027**	1.0	0.3	0.940
**Low Hemoglobin**	3.4	0.6	**0.041**	1.0	0.1	0.995
**Low serum sodium**	1.2	0.2	0.459	-	-	-
**High C-reactive protein**	3.1	0.4	**0.012**	0.9	0.4	0.853
**High RBS**	1.3	0.2	0.361	-	-	-

HCAI, healthcare associated infection; RBS, random blood sugar. Bold values mean significant *p*-values.

### Treatment Outcomes

There were 174 (43.5%) patients with at least one episode of HCAI. The most prevalent HCAI was catheter related bloodstream infection (CRBSI) [64 (36.8%)], while peritonitis [45 (25.8%)] and pneumonia [37 (21.2%)] were also very common. The most frequent treatment outcome seen in this study was cured [113 (64.9%)], followed by death [37 (21.3%)] and treatment failure 17 (9.8%). Moreover, there were 66 (37.9%) patients with recurrent HCAIs. Clinical and sociodemographic variables, such as age, type of dialysis, recurrent HCAIs, type of vascular access, occurrence of CRBSI, sodium and hemoglobin levels were significantly associated with these treatment outcomes **(** Table 4 **)**.

**TABLE 4:** Demographics and clinical variables associated with treatment outcomes of healthcare associated infections.

		Treatment outcomes			
	Patients with HCAIs (n= 174)	Clinical cure (*n* = 113)	Death (*n* = 37)	Treatment failure (*n* = 17)	Defaulted or transferred out (*n* = 7)	*p*-value*
**Median age in years (IQR)**	62 (16)	61 (17)	66 (16)	60 (20)	73 (27)	**0.001**
**Gender**						
Male	86 (49.4%)	53 (46.9 %)	19 (51.4%)	10 (58.8%)	4 (57.1%)	
Female	88 (50.6%)	60 (53.1%)	18 (48.6%)	7 (41.2%)	3 (42.9%)	
**Age group**						**0.298**
18–40 years	16 (9.2%)	12 (10.6%)	2 (5.4%)	2 (11.8%)	0 (0.0%)	
41–60 years	51 (29.3%)	33.6 (30%)	6 (16.2%)	6 (35.3%)	2 (28.6%)	
>60 years	107 (61.5%)	63 (55.8%)	29 (78.4%)	9 (52.9%)	5 (71.4%)	
**Body mass index (kg/m^2^)** * **(n = 145)**						0.690
Under weight (Below 18.5)	12 (8.3%)	8 (8.6%)	4 (12.5%)	0 (0.0%)	0 (0.0%)	
Healthy or normal (18.5 – 24.9)	61 (42.1%)	37 (39.8%)	15 (46.9%)	6 (42.9%)	3 (60%)	
Overweight (25–29.9)	39 (26.9%)	26 (28%)	5 (15.6%)	6 (42.9%)	1 (20%)	
Obese (More than 30)	33 (22.8%)	22 (23.7%)	8 (25%)	2 (14.3%)	1 (20%)	
**Comorbidities**						
Diabetes	123 (70.7%)	80 (70.8%)	23 (62.2%)	14 (82.4%)	5 (71.4%)	0.501
Hypertension	160 (91.95%)	103 (91.2%)	35 (94.6%)	15 (88.2)	7 (100%)	0.701
Hyperlipidemia	38 (21.8%)	21 (18.6%)	10 (27%)	5 (29.4%)	1 (14.3%)	0.553
Ischemic heart disease	36 (20.68%)	24 (21.2%)	7 (18.9%)	2 (11.8%)	3 (42.9)	0.390
Congestive heart failure	15 (8.62%)	13 (11.5%)	1 (2.7%)	1 (5.9%)	0 (0.0%)	0.301
Anemia	13 (7.47%)	9 (8%)	2 (5.4%)	1 (5.9%)	1 (14.3%)	0.849
**Infections recurrence**	66 (37.9%)	34 (30.1%)	14 (37.8%)	16 (94.1%)	2 (28.6%)	**<0.001**
**No. of comorbidities**						0.612
2 or less	67 (38.5%)	45 (39.8%)	15 (40.5%)	4 (23.5%)	3 (42.9%)	
More than 2	107 (61.5%)	68 (60.2%)	22 (59.5%)	13 (76.5%)	4 (57.1%)	
**No. of medications patient was taking**						0.090
Less than 5	2 (1.2%)	0 (0.0%)	2 (5.4%)	0 (0.0%)	0 (0.0%)	
5 to 10	119 (68.8%)	74 (66.5%)	28 (75.7%)	11 (68.8%)	6 (85.7%)	
More than 10	52 (30.1%)	34.5 (31%)	7 (18.9%)	5 (31.3%)	1 (14.3)	
**No. of years on dialysis**						0.382
2 or less	75 (43.1%)	44 (38.9%)	16 (43.2%)	10 (58.8%)	4 (57.1%)	
More than 2	99 (56.9%)	69 (61.1%)	21 (56.8%)	7 (41.2%)	3 (42.9%)	
**Type of dialysis**						**0.039**
Hemodialysis	101 (58%)	65 (57.5%)	18 (48.6%)	15 (88.2%)	3 (42.9%)	
Peritoneal dialysis	73 (42%)	48 (42.5%)	19 (51.4%)	2 (11.8%)	4 (57.1%)	
**Type of vascular access**						**0.003**
Arterioventricular fistula (AVF)	27 (15.5%)	20 (17.9%)	6 (16.2%)	0 (0.0%)	1 (14.3%)	
Central venous catheter (CVC)	47 (26.9%)	24 (21.4%)	8 (21.4%)	12 (70.6%)	2 (28.6%)	
Tenchkoff catheter	69 (39.65%)	44 (39.3%)	19 (51.4%)	2 (11.8%)	4 (57.1%)	
Multiple accesses^a^	31 (17.81%)	24 (21.4%)	4 (10.8%)	3 (17.6%)	0 (0.0%)	
**Type of HCAI infection**						
CRBSI	64 (36.8%)	40 (35.4%)	9 (24.3%)	13 (76.5%)	2 (28.6%)	**0.003**
Peritonitis	45 (25.8%)	29 (25.7%)	12 (32.4%)	1 (5.9%)	3 (42.9%)	0.143
Pneumonia	37 (21.2%)	23 (20.4%)	10 (27%)	3 (17.6%)	0 (0.0%)	0.418
Bacteremia	23 (13.2%)	13 (11.5%)	6 (16.2%)	4 (23.5%)	0 (0.0%)	0.359
Exit site infection	19 (10.9%)	13 (11.5%)	2 (5.4%)	3 (17.6%)	1 (14.3%)	0.558
UTI	6 (3.4%)	2 (1.8%)	3 (8.1%)	1 (5.9%)	0 (0.0%)	0.270
**TWBCs**						0.117
Normal	83 (47.7%)	50 (44.2%)	19 (51.4%)	10 (58.8%)	3 (42.9%)	
High	82 (47.1%)	60 (53.1%)	13 (35.1%)	6 (35.3%)	4 (57.1%)	
Low	9 (5.2%)	3 (2.7%)	5 (13.5%)	1 (5.9%)	0 (0.0%)	
**Hemoglobin**						**<0.001**
Normal	24 (13.8%)	18 (15.9%)	1 (2.7%)	4 (23.5%)	2 (28.6%)	
Low	150 (86.2%)	95 (84.1%)	36 (97.1%)	13 (76.5%)	5 (71.4%)	
**Urea**						**0.048**
High	162 (93.6%)	104 (91.7%)	36 (97.3%)	17 (100%)	5 (71.4%)	
Normal	11 (6.4%)	9 (8.3%)	1 (2.7%)	0 (0.0%)	2 (28.6%)	
**Sodium**						**0.011**
Normal	95 (54.6%)	72 (63.7%)	17 (45.9%)	5 (29.4%)	2 (28.6%)	
Low	79 (45.4%)	41 (36.3%)	20 (54.1%)	12 (70.6%)	5 (71.4%)	
**HbA1c range**						0.213
High	91 (56.5%)	59 (56.2%)	17 (50%)	12 (80%)	3 (42.9%)	
Normal	70 (43.5%)	46 (43.8%)	17 (50%)	3 (20%)	4 (57.1%)	
**Albumin**						0.119
Normal	18 (10.3%)	16 (14.2%)	0 (0.0%)	2 (11.8%)	1 (14.3%)	
Low	156 (89.7%)	97 (85.8%)	37 (100%)	15 (88.2%)	6 (85.7%)	
**Random blood sugar**						0.569
High	75 (57.7%)	46 (54.1%)	18 (66.7%)	8 (61.5%)	2 (40%)	
Normal	55 (42.3%)	39 (45.9%)	9 (33.3%)	5 (38.5%)	3 (60%)	

* Chi square test.

a More than one type of dialysis access used during renal replacement therapy.

IQR, interquartile range; CRBSI, catheter related blood stream infection, UTI: urinary tract infection; TWBCs, total white blood cells; HbA1c, hemoglobin A1c. Bold values mean significant *p*-values.

In univariate multinomial logistic regression, factors such as advancing age (OR = 1.06; 95%CI:1.02–1.1, *p*-value = 0.001), low serum sodium levels (OR = 2; 95%CI:0.9–4.3, *p*-value = 0.059) and low blood hemoglobin concentration (OR = 6.8; 95%CI:0.8–52, *p*-value = 0.066) were significantly associated with higher number of deaths from HCAIs. However, recurrent HCAIs (OR = 37; 95%CI:4.7–291, *p*-value = 0.001), occurrence of CRBSI (OR = 5.9; 95%CI:1.8–19.4, *p*-value≤0.003), use of CVCs (OR = 8.8; 95%CI:2.8–27, *p*-value<0.001), and low serum sodium levels (OR = 4.2; 95%CI:1.3–12.8, *p*-value = 0.011) were more likely to cause treatment failure in patients with HCAIs. During multivariate multinomial logistic regression, advancing age (OR = 1.06; 95%CI:1.02–1.1, *p*-value = 0.001), and low blood hemoglobin concentration (OR = 9.1; 95%CI:1.1–86, *p*-value = 0.039) were found to be independent risk factors for death as a treatment outcome, while recurrent HCAIs (OR = 33; 95%CI:4–282, *p*-value = 0.001), use of CVCs (OR = 4.2; 95%CI:0.9–18.6, *p*-value = 0.053) and low serum sodium levels (OR = 3.9; 95%CI:0.9–15, *p*-value = 0.052) were risk factors associated with treatment failure **(** Table 5 **)**.

**TABLE 5:** Multinomial logistic regression for risk factors associated with unsuccessful treatment outcomes.

	Univariate multinomial logistic regression		Multivariate multinomial logistic regression	
	Odds ratio [95% CI (significance; *p*-value)]		Odds ratio [95% CI (significance; *p*-value)]	
	Death versus cure	Treatment failure vs cure	Death versus cure	Treatment failure vs cure
Age	1.06 [1.02–1.1 (0.001)]	0.99 [0.95–1 (0.887)]	1.06 [1.02–1.1 (0.001)]	0.97 [0.9–1 (0.329)]
Recurrent HCAIs	1.4 [0.6–3 (0.382)]	37 [4.7–291 (0.001)]	1.6 [0.6–3.9 (0.289)]	33 [4–282 (0.001)]
CRBSI	0.5 [0.2–1.3 (0.216)]	5.9 [1.8–19.4 (0.003)]	0.9 [0.3–2.7 (0.913)]	2.9 [0.6–12.6 (0.156)]
Type of dialysis				
Hemodialysis	0.7 [0.3–1.4 (0.347)]	5.5 [1.2–25 (0.027)]	0.4 [0.1–1.2 (0.130)]	2.6 [0.3–18.7 (0.321)]
Peritoneal dialysis	Ref	Ref	Ref	Ref
Type of vascular access				
AVF	0.8 [0.3–2.4 (0.820)]			
CVC	1 [0.4–2.4 (0.980)]	8.8 [2.8–27 (<0.001)]	0.9 [0.2–3.4 (0.913)]	4.2 [0.9–18.6 (0.053)]
Tenchkoff catheter	1.6 [0.7–3.4 (0.200)]	0.2 [0.04–0.9 (0.042)]	1.8 [0.7–4.4 (0.182)]	0.2 [0.03–1 (0.054)]
Multiple accesses	0.4 [0.1–1.3 (0.160)]	0.7 [0.2–2.9 (0.722)]	-	-
Hemoglobin				
Normal	0.1 [0.01–1.1 (0.066)]	1.6 [0.4–5.5 (0.439)]	-	-
Low	6.8 [0.8–52 (0.066)]	0.6 [0.1–2.1 (0.439)]	9.8 [1.1–86 (0.039)]	0.8 [0.1–4.2 (0.809)]
Urea				
High	2.7 [0.3–22.9 (0.345)]	-	-	-
Normal	0.3 [0.04–2.9 (0.345)]	-	-	-
Sodium				
Normal	0.4 [0.2–1 (0.059)]	0.2 [0.07–0.7 (0.011)]	-	-
Low	2 [0.9–4.3 (0.059)]	4.2 [1.3–12.8 (0.011)]	1.6 [0.7–3.7 (0.240)]	3.9 [0.9–15 (0.052)]

HCAIs, healthcare associated infections; CRBSI, catheter related blood stream infections; AVF, Arterioventricular fistula; CVC, central venous catheter.

## Discussion

The study reported a high prevalence (43.5%) of HCAIs among ESRD disease patients undergoing renal RRT. Among the 174 patients with HCAIs, clinical cure (64.9%) was the most frequent treatment outcome, followed by death (21.3%) and treatment failure (9.8%). Patients’ characteristics such as advancing age, low serum sodium levels and low blood Hb concentration were associated with increased mortality in these patients. Furthermore, recurrent HCAIs, occurrence of CRBSI, use of CVCs, and low serum sodium levels were more likely to cause treatment failure in patients with HCAIs. Multivariate logistic regression showed advancing age, and low blood Hb concentration to be independent risk factors for death as a treatment outcome. Recurrent HCAIs, use of CVCs, and hyponatremia were risk factors associated with treatment failure.

Advancing age has shown to increase the chances of mortality by 1.1-folds. Moreover, within age groups, patients aged 18–40 and 41–60 years had significantly higher mean survival time (22.8 and 22.5 months respectively) than patients aged >60 years (20.7 months). Increased mortality is highly associated with poor functional status and aggregation of clinical comorbidities in elderly patients on RRT (Wright, 2015). According to United States Renal Data System (USRDS) registry, nearly 30% of all older adults (≥65 years) on dialysis died in the year 2015 (Wachterman et al., 2019). Therefore, management of RRT related issues such as maintaining vascular access and infection complications, must be addressed with caution. Clinicians must incorporate screening and treatment strategies for various RRT associated complications in elderly patients, including infections, in their routine plan of care.

Low blood Hb concentration was likely to cause 10-folds increase in mortality in the present study. Similarly, a higher mean survival time of 22.9 months was seen in patients with normal blood Hb concentration compared to mean survival time of 21.1 months in patients with lower blood Hb. Similar results have been shown in the previous studies where higher achieved hemoglobin (Hb) levels were associated with decreased mortality (Volkova and Arab, 2006; Bradbury et al., 2009). According to USRDS, longer the patient’s Hb levels remain <11 g/dl, more are the chances of death (Bradbury et al., 2009). The compromised kidney function and prevalence of malnutrition in ESRD patients on RRT, which is evidenced by reduced blood Hb and hypoalbuminemia, may lead to dysfunction of the immune system and therefore, increase the risk of infections induced mortality (Tang and Chen, 2016). Thus, the provision of nutritional support for patients on RRT is vital to help in improving the immune function and decrease infection related deaths. Moreover, adequate dosing and of epoetin alfa (EPO) is necessary to raise Hb concentration in these patients and in case of poor Hb response to EPO therapy, proper dosage adjustment should be performed by pharmacists and physicians.

Patients with recurrent HCAIs were strongly associated with increased occurrence of treatment failure by 33-folds. Another study identified various factors associated with recurrent CRBSI in HD patients, including inappropriate duration of therapy and inappropriate catheter management, while appropriate antibiotic selection was not found to be associated with recurrent CRBSI (Zhang et al., 2019). Due to these recurrent infections, patients on RRT play a predominant role in the epidemics and outbreaks of antibiotic resistant pathogens, such as vancomycin-resistant enterococci (VRE) and vancomycin-resistant *Staphylococcus aureus* (Zacharioudakis et al., 2015). Moreover, first reports on isolation of these resistant pathogens involved patients on RRT (Zacharioudakis et al., 2015). Antimicrobial stewardship programs and infection control and prevention strategies should be implemented to discourage inappropriate use and promote rationale use of antibiotics particularly in dialysis population.

CRBSIs was the most common HCAI found in this study and use of CVCs was associated with 4.2-folds increase in treatment failure as compared to clinical cure. CRBSIs cause considerable increase in morbidity, mortality and healthcare costs (Sahli et al., 2017). Being part of the normal flora, *S. aureus* is the most common pathogen identified in these infections (Sahli et al., 2017), and due to the high prevalence of vancomycin-resistant *S. aureus* in dialysis patients (Zacharioudakis et al., 2015), the chances of treatment failure are considerably high. Empirical therapy with antibiotics against methicillin sensitive *S. aureus* and methicillin resistant *S. aureus* should be considered when there is risk of catheter related infection. Furthermore, microbiological profiles of the patients should be carefully monitored to choose optimum empirical therapy.

In 24 months survival analysis, a significantly higher mean survival time of 22.7 months was seen in patients without HCAIs than of 19.9 months in patients with HCAIs. Infection is one major cause of mortality and increases the length of hospitalization in ESRD patients (McDonald et al., 2014). A study conducted in United States showed that 9.6 percent of all the deaths incident dialysis patients were caused by infections (Collins et al., 2015). Moreover, mortality due to septicemia and sepsis is significantly higher for chronic dialysis patients than in general public (Sarnak and Jaber, 2000). Efforts should be made to develop strict infection control policies and prevention strategies to reduce the risk of HCAIs. The exposure of ESRD patients to hospitals should be minimized to prevent these infections and in turn reduce mortality.

The survival analysis showed that patients with 2 or less comorbidities have shown a comparatively higher mean survival time (22.5 months) than patients with more than 2 comorbidities (20.7 months). Similar results were seen in patients with normal blood sugar levels (22.4 months) compared to patients with higher blood sugar levels (20.6 months). Several studies have reported that better glycemic control is associated with longer survival in patients on RRT, while poor glycemic control increases mortality due to infections (Shurraw et al., 2011; Ricks et al., 2012). Hence, efficient management of glycemic levels can help to reduce the occurrence of HCAIs and improve overall survival.

Hyponatremia has shown to decrease mean survival time in patients on RRT and was also found to cause increase occurrence of treatment failure. Waikar et al. demonstrated a constant connection between hyponatremia and increased mortality on dialysis, and this enhanced mortality is caused partly due to infectious complications (Waikar et al., 2011). Similarly, hyponatremia is correlated with greater risk of infection-related hospitalizations (Mandai et al., 2013). A study suggested that high concentration of serum sodium improve protective action against pathogens by promoting CD4^+^ cells differentiation into T-helper 17 (T_h_17) cells, while hyponatremia inhibits T_h_17 cells (Mandai et al., 2013).

To the best of our knowledge, this is the first study conducted on this topic, however, there were few limitations in our study. Although it was a multicenter study, but the collection of data from only two hospitals limits generalizability of results. To reduce the effect of this limitation to a minimum level, sufficient sample size was used in this study. Secondly, being a retrospective study, only the data present in the patients’ medical records were collected. However, patients record at the hospitals were completed with great care and organization. Additionally, all the laboratory tests were performed using standardized protocols in these hospitals, hence it is unlikely that any event of HCAI remained undiagnosed. Moreover, the authors did their best to make sure that the data was collected with great caution, and all ambiguities were resolved by the help of hospital staff and co-investigators from the hospitals. The third limitation of this study is related to the search of patients. All patients were searched using ICD codes for ESRD, hence there is a chance of skipping any patient that fulfilled the criteria for inclusion but was diagnosed with different ICD code. However, random sampling technique was used in this study to overcome this problem. Finally, the modified version of Friedman’s definition of HCAIs was used in this study. It is possible that using any other definition might affect the results.

## Conclusion

The high burden of HCAIs is profound challenge faced by patients on RRT, which not only effects the treatment outcomes but also contribute substantially to the poor survival among these patients. Infection control policies and prevention strategies should be implemented to overcome these risk factors. Recurrent infections and use of antibiotics are considerably high among the patients on RRT due to frequent and complex surgical procedures and various comorbid conditions. Therefore, appropriate selection, duration, and use of antibiotics is pivotal to not only improve patients’ clinical outcomes but also to decrease the prevalence antibiotic resistant pathogens. Patient contact with hospitals and other healthcare settings should be kept to minimum to reduce the risk of infections, and hence improving clinical outcomes and survival among ESRD patients. Future prospective studies and randomized control trials should provide a narrow focus on antibiotic therapy, treatment outcomes and their associated risk factors, which will further clarify the burden and effect of these contributing factors.

## Data Availability

The raw data supporting the conclusions of this article will be made available by the authors, without undue reservation.
